# Strategy-Specific Patterns of *Arc* Expression in the Retrosplenial Cortex and Hippocampus during T-Maze Learning in Rats

**DOI:** 10.3390/brainsci10110854

**Published:** 2020-11-13

**Authors:** Rafał Czajkowski, Bartosz Zglinicki, Emilia Rejmak, Witold Konopka

**Affiliations:** 1 Laboratory of Spatial Memory, Nencki Institute of Experimental Biology, 02-093 Warsaw, Poland; 2 Neurobiology Center, Nencki Institute of Experimental Biology, 02-093 Warsaw, Poland; bartosz.zglinicki@gmail.com (B.Z.); w.konopka@nencki.edu.pl (W.K.); 3 Laboratory of Neurobiology, BRAINCITY, Nencki Institute of Experimental Biology, 02-093 Warsaw, Poland; e.rejmak@nencki.edu.pl

**Keywords:** retrosplenial cortex, hippocampus, T maze, fluorescence in situ hybridization, *Arc*, spatial memory, rat

## Abstract

The retrosplenial cortex (RSC) belongs to the spatial memory circuit, but the precise timeline of its involvement and the relation to hippocampal activation have not been sufficiently described. We trained rats in a modified version of the T maze with transparent walls and distant visual cues to induce the formation of allocentric spatial memory. We used two distinct salient contexts associated with opposite sequences of turns. Switching between contexts allowed us to test the ability of animals to utilize spatial information. We then applied a CatFISH approach with a probe directed against the *Arc* immediate early gene in order to visualize the associated memory engrams in the RSC and the hippocampus. After training, rats displayed two strategies to solve the maze, with half of the animals relying on distant spatial cues (allocentric) and the other half using egocentric strategy. Rats that did not utilize the spatial cues showed higher *Arc* levels in the RSC compared to the allocentric group. The overlap between the two context engrams in the RSC was similar in both groups. These results show differential involvement of the RSC and hippocampus during spatial memory acquisition and point toward their distinct roles in forming the cognitive maps.

## 1. Introduction

The ability to efficiently and flexibly navigate a complex environment is one of the most demanding functions of each nonsedentary organism. In mammals, the central structure responsible for this type of memory is the hippocampus, where the cognitive map is stored. Other cortical and subcortical structures facilitate the formation of the spatial map and together with the hippocampus they form an interconnected network that serves spatial navigation [1]. The retrosplenial cortex (RSC) has long been considered one of the relatively less important components of the spatial memory circuit. The research on this structure accelerated rapidly over the course of last decade [2,3]. The connectivity of the RSC strongly supports its involvement in the spatial memory circuit. It receives input from the hippocampus and projects to the medial entorhinal cortex [4] and pre- and parasubiculum [5]. Notably, the RSC contains head direction cells [6]. Interestingly, the firing patterns of these neurons not only reflect the passive head turns but also respond to complex spatial relationships related to the internal and external reference frame [7,8,9]. They are also capable of encoding the reward location [10,11]. Evidence exists of learning-related plasticity within this structure [12], a process that may result in the emergence of a cortical memory engram [13]. Neurons involved in this engram are capable of supporting contextual memory even without the involvement of the hippocampus [14]. It is therefore well documented in both animal models and human studies that the RSC is involved in orientation and navigation based on distant visual landmarks. The exact timeline of RSC involvement in the acquisition of this type of memory and its temporal correlation with formation of the spatial strategy have been relatively poorly studied. It is also not known whether the RSC is capable of encoding orthogonal representations of the context, similarly to the hippocampus. Rats may spontaneously develop a number of navigational strategies that can be based either on local (egocentric) or external (allocentric) cues. These behaviors can be measured and quantified under controllable conditions, making the rat model an ideal choice for a study of the relationship between the activity of a given brain region and the behavioral output. The goal of our study was to identify the behavioral strategies of rats in a visually guided version of a navigation-based task (T maze) and to compare the activity of retrosplenial cortex and hippocampus after learning under the allocentric and egocentric strategies. Fluorescence in situ histochemistry (FISH) for the immediate early gene *Arc* was used to map the learning-induced plasticity within the RSC. This method has previously been used to reveal the memory engram in several other behavioral paradigms related to spatial learning [15].

## 2. Materials and Methods

### 2.1. Animals

Fourteen young (3 months old) Wistar rats were used for the experiment (maximum number of animals that allowed completion of early training sessions within a timeframe of 12 h). Rats were kept on a 12 h light/dark cycle. The experimental protocol followed the European Communities Council Directive and The Law on the Protection of Animals Used for Scientific or Educational Purposes and was approved by the 1st Local Ethical Committee in Warsaw (code: 496/2013). Experiments were conducted during the light phase. Before the experiment, food and water were available ad libitum. Ten days prior to the onset of training, rats were separated into single cages, and the average daily calorie intake was monitored for each animal. During the subsequent phases the amount of regular feed was adjusted in order to account for additional calories provided by food reward. Five days before the onset of training, rats were accustomed to a Cheerio food reward (16 pieces per day). Rats were handled for 10 days prior to the onset of training. Handling sessions included transport from holding room to the experimental room and individual handling for 2 min. One day before the training phase, the rats were exposed to the experimental apparatus during a 5 min exploration session.

### 2.2. Behavioral Training

**Figure 1:**
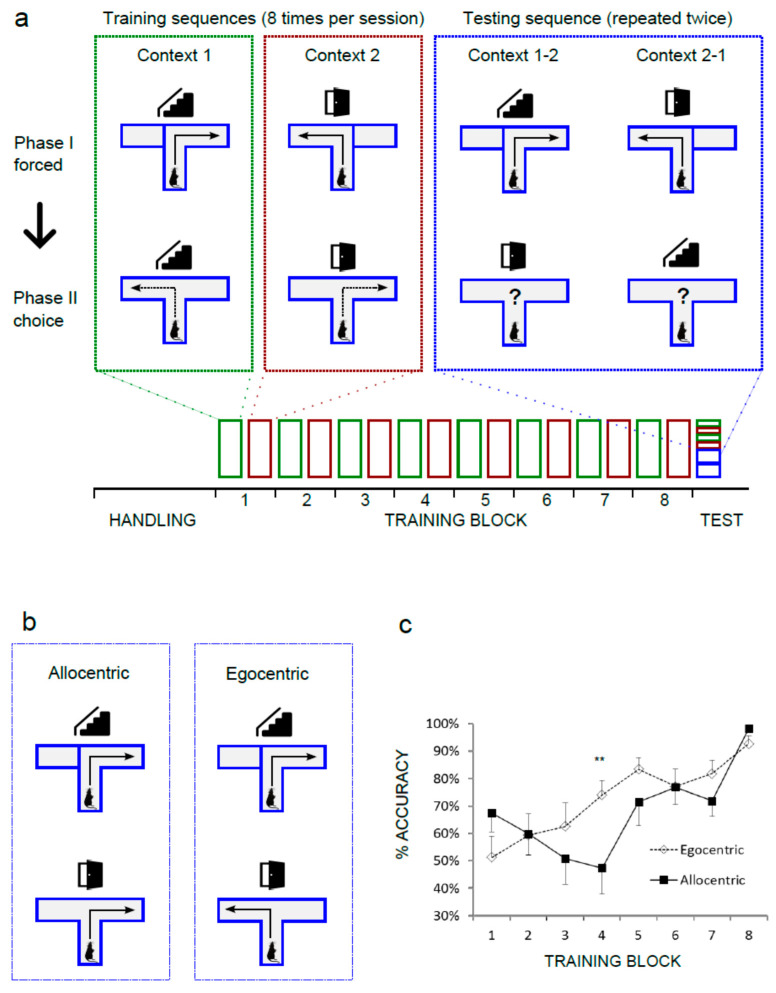
Rats learn to alternate in the “win–shift” version of the T maze using two strategies. (**a**) Simplified schematic diagram of the behavioral protocol. Rectangles represent training sessions or testing trials. Icons represent two spatial contexts (photos of the Nencki Institute interiors): “stair” and “door”. (**b**) Two possible navigational outcomes during the context switching test. (**c**) Learning curves for the “allocentric” group, *n* = 7, and “egocentric” group, *n* = 6. Significant effect: strategy × session block, (**) *p* < 0.01. Bars represent ± SEM.

The standard T-maze design was modified in order to achieve higher dependence on allothetic, extra-maze cues and to promote the acquisition of behavioral strategy based on these cues. Opaque walls were replaced by transparent Plexiglas. A digital projector was used to display two distinct images of Nencki Institute interiors (Context A and Context B) in front of the maze. Both images were adjusted for equal overall brightness and RGB levels. The experimental design is summarized in Figure 1a. Each behavioral trial consisted of a forced run, with one arm blocked and the open arm rewarded, immediately followed by a choice run with both arms open but with reward only in previously unvisited arm (“win–shift” strategy). Each animal was subjected to 8 trials per day, with a 2 min inter-trial interval. On each day, only one context was used. Each context required a different sequence of turns during the forced and the choice phase. For CtxA (“stair”), the sequence was right/left, and for CtxB (“door”), it was reversed (left/right). Contexts were changed daily for 16 days. On days 1–8, one Cheerio loop was used as reward, and on days 9–16, half a loop was used. Each choice trial was scored as correct when the animal entered the correct arm and collected the food reward. Other cases were counted as incorrect, including trials where the animal entered an arm (as defined by crossing the entrance with the tail base) and withdrew before reaching the end. After each failed trial, the animal was immediately removed from the maze. During the test day, the animals were first subjected to four control trials (two in each context, alternating). For the strategy assessment, animals performed another four trials with the context changed during the choice phase. Both arms were rewarded during that phase in order to avoid memory extinction. Rats were classified into the “allocentric” or “egocentric” group on the basis of their performance during four test trials. The threshold was estimated based on the average probability of error in the last training block, where animals reached asymptotic performance (Figure 1c). In that block, the animals completed a total of 199/208 correct trials, and the frequency of error (lack of alternation between sample phase and choice phase) was 0.043. Under these assumptions, the following probabilities of alternations were calculated for the test session: 4/4, *p* = 0.839; 3/4, *p* = 0.15; 2/4, *p* = 0.01; 1/4, *p* = 0.0003; 0/4, *p* = 0.000003. The animals that performed two or less alternations in four test trials were therefore considered allocentric.

### 2.3. Fluorescence In Situ Hybridization for Arc Immediate Early Gene (CatFISH)

**Figure 2:**
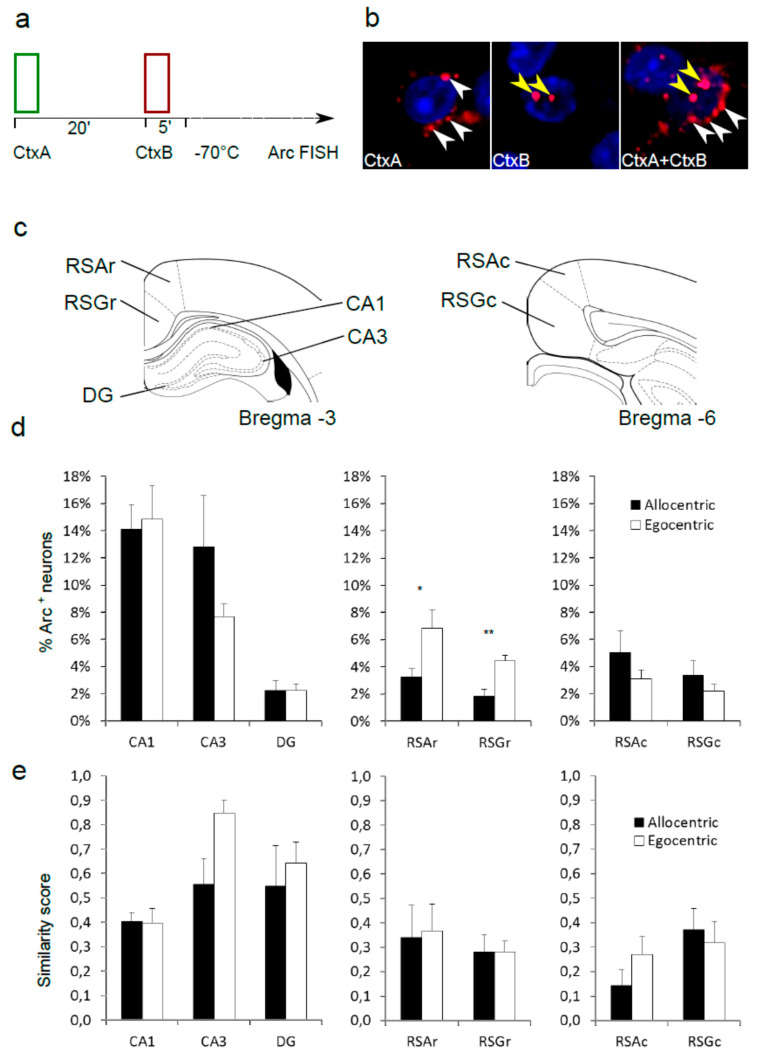
Immediate early gene *Arc* mRNA expression after T maze sessions in two spatial contexts differs between “allocentric” and “egocentric” rats. (**a**) Schematic diagram of experimental procedure. (**b**) Sample images of *Arc* mRNA in situ hybridization detected in cytoplasm (triggered by Context A, white arrowheads), nucleus (induced by Context B, yellow arrowheads) or in both cellular compartments. (**c**) Localization of anatomical regions selected for *Arc* quantification. (**d**) Percentage of *Arc*-positive nuclei (triggered by Context B) in the dorsal hippocampus and rostral and caudal retrosplenial cortex (RSC). Significant effects: (*) rostral RSA, *q* < 0.1; (**) rostral RSG, *q* < 0.05 *t*-test with FDR adjustment. (**e**) Colocalization of the nuclear and cytoplasmic *Arc* probe after sequential sessions in two contexts.

Before the CatFISH experiment, animals were allowed to rest for 48 h after the strategy test session. In order to induce *Arc*, each animal was subjected to a single trial in CtxA, followed by one trial in CtxB, with a 20 min inter-trial interval (Figure 2a). Two minutes after the beginning of the second trial, each animal was quickly sedated with isoflurane and decapitated with a guillotine. Brains were quickly removed and flash-frozen in −50 °C isopentane. After embedding in OCT medium (Leica), brains were cryosectioned into 20 µm slices on a cryostat (Leica) and placed on Superfrost slides. Slides were stored at −80 °C. Fluorescence in situ hybridization was performed as described previously [15,16]. The coding sequence of *Arc* was reverse transcribed and cloned into a BlueScript plasmid. Fluorescein-labeled riboprobes were generated from the plasmid with T3 or T7 polymerase sites. Brain sections were fixed in 4% paraformaldehyde, washed in phosphate buffered saline (PBS), and incubated in 1% H_2_O_2_ for 20 min. An acetylation buffer (0.1 M triethanolamine, 0.25% HCl, 0.25% acetic anhydride) was applied for 15 min at room temperature. Next, brain sections were prehybridized for 3 h in a prehybridization solution (Sigma-Aldrich, Waltham, MA, USA), followed by overnight hybridization at 65 °C in a hybridization solution (Sigma-Aldrich, Waltham, MA, USA) containing sense or antisense probes. Afterward, brain sections were washed in 0.5 × saline-sodium citrate (SSC). Then, TNB blocking solution (TSA Plus System; PerkinElmer Life and Analytical Sciences, Turku, Finland) was applied for 1 h. Brain sections were incubated with anti-fluorescein antibody (Fab fragments) coupled to horseradish peroxidase (1:200; Roche Applied Science, Penzberg, Germany) overnight in 4 °C, washed with PBS with 0.1% Triton X-100 (PBST). The hybridization signal was amplified with the Cy3 TSA Plus System (PerkinElmer Life and Analytical Sciences, Turku, Finland). Slides were imaged with a Zeiss Spinning Disc microscope (Carl Zeiss, Jena, Germany). A 40 × /1.20 C apochromatic water immersion objective was used. DAPI was excited with a 405 nm diode laser and imaged with a 442/46 nm emission filter. Cy3 was excited with a 561 nm diode laser and imaged with a 525/50 nm emission filter. Image stacks containing the entire slice thickness were acquired with a Z interval of 1 µm. Only middle sections containing whole cells were used for analysis. FIJI software (v1.51r, NIH, Bethesda, MD, USA) was used for thresholding the images (Otsu method) and for manual cell counting. DAPI labeling was first used to obtain the total cell count for each image. Individual neurons were then analyzed for the presence of the *Arc* probe within the nuclear region defined by DAPI staining. Finally, each neuron with confirmed nuclear *Arc* labeling was analyzed for the presence of an adjacent cytoplasmic *Arc* probe. Nuclei from three adjacent brain slices were counted for each region. All neuronal layers were included in the analysis in the RSC. The granular cell layer was counted in dentate gyrus (DG), pyramidal cell layers were counted in CA1 and CA3 fields. The average number of counted nuclei per slice were as follows: retrosplenial agranular (RSA) rostral: 224 ± 29, retrosplenial granular (RSG) rostral: 321 ± 26, CA1: 106 ± 10, CA3: 50 ± 5, DG: 122 ± 48, RSA caudal: 318 ± 37, and RSG caudal: 260 ± 25. *Arc* expression data were presented as the percentage of *Arc*-positive nuclei per total number of neurons. *Arc* overlap between CtxA and CtxB was calculated using similarity score [17]. All analyses were done blind.

### 2.4. Data Processing

For statistical analysis, we used Prism 7.01 software (GraphPad Software Inc., San Diego, CA, USA). To compare learning between the allocentric vs. the egocentric group, two-way repeated-measures ANOVA was applied, with post hoc Bonferroni test to analyze individual time points. To compare *Arc* expression levels and the overlap scores between behavioral groups in selected brain areas, multiple *t*-test was used with false discovery rate (FDR) adjustment according to the two-stage linear step-up procedure of Benjamini, Krieger, and Yekutieli, with Q = 10% assumed.

## 3. Results

### 3.1. Behavioral Strategies in T-Maze Task

In order to assess the behavioral strategy used by rats in the spatially guided T maze we first trained a cohort of animals to perform the task with high precision. Animals were expected to reach at least 87.5% accuracy, with each rat correctly completing a minimum of 14 out of 16 trials in each session block (one session in CtxA and one in CtxB). In all, 13 out of 14 rats learned to alternate between the forced and choice phase in both contexts and exceeded the criterion by Session 8 (Figure 1a,c). One animal was excluded. On the test day, each animal was first subjected to four standard trials in order to confirm the memory retention and to control for the effect of novel arrangements of CtxA and CtxB (Figure 1a). All animals completed these trials with 100% accuracy. Next, animals completed four test trials with contexts switched between forced and choice phase (Figure 1a). Depending on the strategy, two outcomes were possible. In the case of an egocentric strategy, the animal did not react to the context change and entered the opposite arm in the choice run. If an allocentric strategy was used, the animal reentered the previously visited arm, in contingency with the trial order and the context presented (Figure 1b). The preferred strategy of each rat was determined by the number of errors during four test trials (see Methods, Section 2.2 for details). The natural tendency of rodents that develops in the absence of external cues (“win–shift” strategy), is to alternate and to explore the previously unvisited arm. It does not require any external reference. Animals that followed the alternation pattern and ignored the contexts in at least three trials were therefore considered “egocentric”. Animals that followed the learned spatial context and revisited the arm at least in two test trials were counted as “allocentric”. Out of 13 animals, seven rats used a predominantly allocentric strategy, whereas six made no more than one re-entry, suggesting an egocentric, context-independent strategy. We did a post hoc analysis of the learning curves for both groups. In Figure 1c, results for each session block were plotted as percentage of correct choice trials for the groups later identified as “allocentric” and “egocentric”. Comparison of performance between allocentric and egocentric groups (two-way repeated-measures ANOVA) revealed the significant effect of session number (F(7,77) = 12.06, *p* < 0.0001), and an interaction between session number and strategy (F(7,77) = 3.201, *p* = 0.0050). Post hoc analysis (Bonferroni) showed significant difference between groups at session block 4 (adjusted *p* = 0.0094).

### 3.2. CatFISH

After a 48 h delay, the same cohort of rats was subjected to two test trials, one in each context, instantly followed by brain extraction. These trials were separated by 20 min in order to capture *Arc* mRNA at different stages of processing (Figure 2a, see Methods, Section 2.3 for details). All rats completed both trials with no errors, and there was no observable behavioral difference between the groups defined as allocentric and egocentric in the previous experiment. Despite the lack of behavioral differences, comparison between the groups revealed differences in *Arc* mRNA expression within the rostral part of the retrosplenial cortex. Analysis of nuclear *Arc* (triggered by the second trial) revealed that in both the granular (RSA) and the agranular part (RSG) the expression was markedly higher in the group that previously did not use allocentric strategy during the behavioral test (*p* = 0.027, *q* = 0.089 for RSA, *p* = 0.002 and for RSG, *q* = 0.012 *t*-test with FDR adjustment) (Figure 2a). In the caudal part of the RSC, the *Arc* expression was similar for both groups, although a reverse tendency could be observed compared to that in the rostral part, with more *Arc* expression in allocentric animals. This trend did not reach statistical significance. Importantly, the expression in all subfields of the dorsal hippocampus was similar for both allocentric and egocentric animals.

The overlap score between the population of neurons with cytoplasmic localization of the *Arc* probe (triggered during first trial) and the cells with nuclear *Arc* (activated during second trial) was used to estimate the similarity between neuronal ensembles activated by both trials [17]. For the hippocampus, abundant functional data about spatial processing exist [18]. We expected the CA3 region to show a relatively high score for the animals that failed to separate spatial contexts (a measure of high pattern completion). Indeed, the egocentric group showed a trend toward a higher degree of overlap (similarity score = 0.85), while the similarity for the allocentric group was lower (similarity score = 0.55, *p* = 0.037, *t*-test). This difference did not retain statistical significance when corrected for the false discovery rate of the experiment (*q* = 0.306). For all regions of the RSC, the similarity scores were not dependent on strategy (Figure 2e).

## 4. Discussion and Conclusions

Over the last decade, the retrosplenial cortex has emerged as a key anatomical structure in the formation of spatial memory in mammals [3,19]. Clinical case studies and functional imaging in humans revealed its role in route planning [20,21], as well as in location and orientation within broader environment [22]. Three lines of investigation were pursued in order to fully reveal the complex involvement of the RSC in rodent models. Electrophysiological recordings showed a complex, multimodal system of spatially tuned neurons in the RSC that respond to different combinations of spatial cues [7,8,9,10,11,23]. Lesion studies progressed from finely positioned electrolytic interventions [24,25,26] to temporarily restricted pharmacological inactivation studies [12,27] and ended with an optogenetic approach [14]. Finally, immediate early gene studies started to reveal the map of learning-induced plasticity within the structures responsible for spatial memory. This third approach currently allows to visualize the progression of the memory formation process [12,13,28,29] as well as anatomical differences within the structures involved [30,31]. Our study contributes to the existing body of work by correlating the immediate early genes (IEG) activation with behavioral strategy assumed by rats during the execution of a spatial task.

We found out that even in a genetically homogenous group of experimental rats, at least two strategies for solving the task could be observed after a period of learning. Importantly, the measured behavioral performance of “allocentric” and “egocentric” animals was indistinguishable at the time of the last four blocks of training, during control trials, and during the CatFISH trials. The only observable difference occurred at the relatively early stage of learning. On session block 4, the animals that later tested as allocentric still showed performance at the level of chance (Figure 1c). Interestingly, the preferred innate strategy of rats is to promptly start alternating between sample and test phase [32]. This mechanism is reflected in the shape of the learning curve for the “egocentric” group. It is therefore reasonable to speculate that the allocentric strategy is starting to develop in some animals around those early time points. Since this process is not driven by the reward (reward frequency remains low until new strategy emerges) we may assume that other mechanisms contribute. It is beyond the scope of this study to infer about the possible circuits. Individual differences in eyesight, visual processing, attention, or short-term spatial memory are possible candidates.

The levels of *Arc* mRNA expression reflect the ongoing plasticity of brain circuits during memory formation. The two behavioral strategies could be associated with differences in forming or utilizing the internal representation of the context (cognitive map). The egocentric animals appear to be at a different stage of spatial learning, either exploring and encoding the layout of the environment or learning the rules between context and behavior. In such a case, more plasticity (as defined by *Arc* expression) occurs. Animals that clearly react to environmental cues by amending their behavior show lower *Arc* expression than the group following the egocentric strategy. This difference could reflect a temporal gradient of RSC plasticity during the acquisition of the spatial task. Apart from temporal differences, an anatomical gradient of *Arc* activation could be observed along the rostro-caudal axis of RSC. This suggests that different subdivisions of the RSC might engage during subsequent stages of learning and memory consolidation [28,29].

The CatFISH approach allows a quantitative comparison between two neuronal ensembles that represent different spatial contexts [15,16]. As expected, in the CA3 region of the hippocampus, we observed a trend toward higher overlap between the two representations of environmental contexts used, but only in the group of rats that failed to use these contexts to guide the behavior, the egocentric group. In the group of allocentric rats, the overlap was much smaller, suggesting the emergence of two separate representations for both contexts. In the RSC, the degree of overlap was comparable in each behavioral group. This suggests that orthogonal representations for diverse contexts are encoded mostly in the hippocampus, while the RSC serves a different role in spatial navigation, possibly encoding more general, schematic representations of the external environment [29].

The CatFISH technique offers an improved insight into the memory circuit compared to classical immediate early gene studies. Still, it can only offer a snapshot of the ongoing learning process. In order to provide a full picture, a number of control studies and time points would be necessary. Beyond the snapshot, this body of work provides a behavioral and conceptual framework for further studies. It does so by demonstrating a behavioral approach to test spatial memory with a within-group control. It appears to be particularly suited for longitudinal approaches, including chronic in vivo IEG imaging. These techniques could be easily implemented in order to expose the missing anatomical and temporal gradients.

## References

[1] Witter M.P., Canto C.B., Couey J.J., Koganezawa N., O′Reilly K.C. (2014). Architecture of spatial circuits in the hippocampal region. Philos. Trans. R. Soc. Lond. B Biol. Sci..

[2] Milczarek M.M., Vann S.D. (2020). The retrosplenial cortex and long-term spatial memory: From the cell to the network. Curr. Opin. Behav. Sci..

[3] Mitchell A.S., Czajkowski R., Zhang N., Jeffery K., Nelson A.J.D. (2018). Retrosplenial cortex and its role in spatial cognition. Brain Neurosci. Adv..

[4] Czajkowski R., Sugar J., Zhang S.J., Couey J.J., Ye J., Witter M.P. (2013). Superficially projecting principal neurons in layer V of medial entorhinal cortex in the rat receive excitatory retrosplenial input. J. Neurosci..

[5] Kononenko N.L., Witter M.P. (2012). Presubiculum layer III conveys retrosplenial input to the medial entorhinal cortex. Hippocampus.

[6] Chen L.L., Lin L.H., Green E.J., Barnes C.A., McNaughton B.L. (1994). Head-direction cells in the rat posterior cortex. I. Anatomical distribution and behavioral modulation. Exp. Brain Res..

[7] Jacob P.Y., Casali G., Spieser L., Page H., Overington D., Jeffery K. (2017). An independent, landmark-dominated head-direction signal in dysgranular retrosplenial cortex. Nat. Neurosci..

[8] Alexander A.S., Nitz D.A. (2015). Retrosplenial cortex maps the conjunction of internal and external spaces. Nat. Neurosci..

[9] Alexander A.S., Nitz D.A. (2017). Spatially Periodic Activation Patterns of Retrosplenial Cortex Encode Route Sub-spaces and Distance Traveled. Curr. Biol..

[10] Mao D., Kandler S., McNaughton B.L., Bonin V. (2017). Sparse orthogonal population representation of spatial context in the retrosplenial cortex. Nat. Commun..

[11] Vedder L.C., Miller A.M., Harrison M.B., Smith D.M. (2016). Retrosplenial Cortical Neurons Encode Navigational Cues, Trajectories and Reward Locations During Goal Directed Navigation. Cereb. Cortex.

[12] Czajkowski R., Jayaprakash B., Wiltgen B., Rogerson T., Guzman-Karlsson M.C., Barth A.L., Trachtenberg J.T., Silva A.J. (2014). Encoding and storage of spatial information in the retrosplenial cortex. Proc. Natl. Acad. Sci. USA.

[13] Milczarek M.M., Vann S.D., Sengpiel F. (2018). Spatial Memory Engram in the Mouse Retrosplenial Cortex. Curr. Biol..

[14] Cowansage K.K., Shuman T., Dillingham B.C., Chang A., Golshani P., Mayford M. (2014). Direct reactivation of a coherent neocortical memory of context. Neuron.

[15] Guzowski J.F., Worley P.F. (2001). Cellular compartment analysis of temporal activity by fluorescence in situ hybridization (catFISH). Curr. Protoc. Neurosci..

[16] Guzowski J.F., McNaughton B.L., Barnes C.A., Worley P.F. (2001). Imaging neural activity with temporal and cellular resolution using FISH. Curr. Opin. Neurobiol..

[17] Kubik S., Buchtova H., Vales K., Stuchlik A. (2014). MK-801 Impairs Cognitive Coordination on a Rotating Arena (Carousel) and Contextual Specificity of Hippocampal Immediate-Early Gene Expression in a Rat Model of Psychosis. Front. Behav. Neurosci..

[18] Yassa M.A., Stark C.E. (2011). Pattern separation in the hippocampus. Trends Neurosci..

[19] Vann S.D., Aggleton J.P., Maguire E.A. (2009). What does the retrosplenial cortex do?. Nat. Rev. Neurosci..

[20] Spiers H.J., Maguire E.A. (2006). Thoughts, behaviour, and brain dynamics during navigation in the real world. Neuroimage.

[21] Maguire E.A. (2001). The retrosplenial contribution to human navigation: A review of lesion and neuroimaging findings. Scand. J. Psychol..

[22] Epstein R.A., Parker W.E., Feiler A.M. (2007). Where am I now? Distinct roles for parahippocampal and retrosplenial cortices in place recognition. J. Neurosci..

[23] Cho J., Sharp P.E. (2001). Head direction, place, and movement correlates for cells in the rat retrosplenial cortex. Behav. Neurosci..

[24] Whishaw I.Q., Maaswinkel H., Gonzalez C.L., Kolb B. (2001). Deficits in allothetic and idiothetic spatial behavior in rats with posterior cingulate cortex lesions. Behav. Brain Res..

[25] Vann S.D., Aggleton J.P. (2004). Testing the importance of the retrosplenial guidance system: Effects of different sized retrosplenial cortex lesions on heading direction and spatial working memory. Behav. Brain Res..

[26] Pothuizen H.H., Aggleton J.P., Vann S.D. (2008). Do rats with retrosplenial cortex lesions lack direction?. Eur. J. Neurosci..

[27] Cooper B.G., Mizumori S.J. (2001). Temporary inactivation of the retrosplenial cortex causes a transient reorganization of spatial coding in the hippocampus. J. Neurosci..

[28] Maviel T., Durkin T.P., Menzaghi F., Bontempi B. (2004). Sites of neocortical reorganization critical for remote spatial memory. Science.

[29] Tse D., Takeuchi T., Kakeyama M., Kajii Y., Okuno H., Tohyama C., Bito H., Morris R.G. (2011). Schema-dependent gene activation and memory encoding in neocortex. Science.

[30] Malinowska M., Niewiadomska M., Wesierska M. (2016). Spatial memory formation differentially affects c-Fos expression in retrosplenial areas during place avoidance training in rats. Acta Neurobiol. Exp..

[31] Vann S.D., Brown M.W., Erichsen J.T., Aggleton J.P. (2000). Fos imaging reveals differential patterns of hippocampal and parahippocampal subfield activation in rats in response to different spatial memory tests. J. Neurosci..

[32] Dudchenko P.A. (2001). How do animals actually solve the T maze?. Behav. Neurosci..

